# Supplementary material for: Development of an Inactivated Camelpox Vaccine from Attenuated Camelpox Virus Strain: Safety and Protection in Camels
Source: Animals (Basel). 2023 Apr 30;13(9):1513. doi: 10.3390/ani13091513 (PMC10177572; doi:10.3390/ani13091513)
Supplement: Supplementary file 1 [file animals-13-01513-s001.zip › animals-2298814-supplementary.pdf]

In order to determine the antibody titer, the highest dilution of the serum that inhibits the development of viral cytopathic effect (CPE) in no less than 50% of infected cell cultures is considered. A scheme of the neutralization reaction and titration of virus-neutralizing antibodies (using a constant virus dose of 100 TCID50) is presented in Table 1. In the given example, the antibody titer is 1:8. The obtained titer (the highest dilution of serum that neutralized the virus in 50% of cell cultures) can be converted from its numerical value to a logarithmic value of 2 using Table 2. Thus, a titer of 1:8 would be equivalent to 3.0 log2.

Table S1. The titration of virus-neutralizing antibodies (with a constant virus dose of 100 TCID50)

	Serum dilution							Contols		
	1:2	1:4	**1:8**	1:16	1:32	1:64	1:128	Virus (100 TCID50) + DMEM	Serum 1:2 + DMEM	DMEM
Serum + virus (100 TCID50)	**-**	**-**	-	+	+	+	+	+	**-**	**-**
	**-**	**-**	-	+	+	+	+	+	**-**	**-**
	**-**	**-**	**+**	+	+	+	+	+	**-**	**-**
	**-**	**-**	**+**	+	+	+	+	+	**-**	**-**
(-) – negative CPE; (+) – positive CPE.										

Table S2. The dilution degree at 1:2 multiplicity in logarithms with bases 2 and 10 is described by Syurin, V.N. in "Guidelines for veterinary virology" (Moscow, Kolos Publishers, 1966, p. 687).

Dilution	Inverse numeric values	Logarithm	
		base 2	base 10
1:2	2,0	1,0	0,3010
1:4	4,0	2,0	0,6021
**1:8**	**8,0**	**3,0**	0,9031
1:16	16,0	4,0	1,2041
1:32	32,0	5,0	1,5051
1:64	64,0	6,0	1,8062
1:128	128,0	7,0	2,1072
1:256	256,0	8,0	2,4082
1:512	512,0	9,0	2,7093
1:1024	1024,0	10,0	3,0103
1:2048	2048,0	11,0	3,3113
